# An annotated T2-weighted magnetic resonance image collection of testicular germ and non-germ cell tumors

**DOI:** 10.1038/s41597-021-00990-z

**Published:** 2021-08-05

**Authors:** Giacomo Feliciani, Lorenzo Mellini, Emiliano Loi, Filippo Piccinini, Roberto Galeotti, Anna Sarnelli, Gian Carlo Parenti

**Affiliations:** 1 IRCCS Istituto Romagnolo per lo Studio dei Tumori (IRST) “Dino Amadori”, Via P. Maroncelli 40, 47014 Meldola (FC), Italy; 2 Department of Morphology, Surgery and Experimental Medicine, University of Ferrara, Via L. Ariosto 34, I-44121 Ferrara, Italy

**Keywords:** Cancer imaging, Testicular cancer

## Abstract

Testicular cancer is a rare tumor with a worldwide incidence that has increased over the last few decades. The majority of these tumors are testicular non-germ (TNGCTs) and germ cell tumors (TGCTs); the latter divided into two broad classes - seminomatous (SGCTs) and non-seminomatous germ cell tumors (NSGCTs). Although ultrasonography (US) maintains a primary role in the diagnostic workup of scrotal pathology, magnetic resonance imaging (MRI) has emerged as the imaging modality recommended for challenging cases, providing additional information to clarify inconclusive/equivocal US. In this work we describe and publicly share a collection of 44 images of annotated T2-weighted MRI lesions from 42 patients. Given that testicular cancer is a rare tumor, we are confident that this collection can be used to validate statistical models and to further investigate TNGCT and TGCT peculiarities using medical imaging features.

## Abstract (summary)

**Table:** 

Measurement(s)	sex cord-gonadal stromal tumor
Technology Type(s)	magnetic resonance imaging
Sample Characteristic - Organism	Homo sapiens

Machine-accessible metadata file describing the reported data: 10.6084/m9.figshare.14511624

## Background & Summary

Although testicular neoplasms are classified as rare tumors, their incidence has increased worldwide in recent years^1^. The malignancy is common among men aged 15–44 years in the U.S., with almost 9600 new cases diagnosed in 2019^2^.These tumors are classified into two categories: (*a*) testicular non-germ cell tumors (TNGCTs) and (*b*) testicular germ cell tumors (TGCTs), the latter divided into two broad classes: (*b1*) seminomatous germ cell tumors (SGCTs) and (*b2*) non-seminomatous germ cell tumors (NSGCTs). It is worthy of note that TGCTs in young men represent the vast majority of testicular neoplasms (nearly 95%), with benign sex cord-stromal tumors accounting for the remaining 5%^3^.

Treatment and prognosis may change on the basis of the above categorization^4^. Advances in treatments, including surgery, chemotherapy and radiation, have resulted in a substantial decrease in the mortality rate of patients with testicular cancer, especially when diagnosed in the early phases.

Color-doppler and conventional ultrasonography (US) are still considered the gold standard for the diagnosis of scrotal pathology, but magnetic resonance imaging (MRI) has emerged as a supplemental imaging modality and is mainly recommended as a problem-solving tool for challenging cases^5^. The final goal of MRI investigations is to reduce the incidence of unnecessary surgery^6^. Previous studies have underlined the role of qualitative radiological assessment based on T1- and T2-weighted MR images, which helps to differentiate between SGCT and NSGCT^7,8^. Furthermore, quantitative diffusion-weighted imaging (DWI) has been shown to have similar accuracy in discriminating between SGCT and NSGCT.

Given the low incidence of these tumors, our dataset could help radiologists to gain valuable experience in recognizing testicular malignancies by visual inspection. Indeed, the dataset is enhanced by orchiectomy-confirmed final diagnosis and a consensus-based MRI visual assessment performed by two expert radiologists. Furthermore, in the past decade, breakthroughs in artificial intelligence (AI) have accelerated the application of computer-based analysis in medical imaging to guide/support clinical decision-making. The present dataset was collected with the main objective of the extraction of quantitative features from digital images, which can provide information not possible from human interpretation alone^9,10^. In a recent publication, Feliciani *et al*.^11^ developed two imaging feature-based models to differentiate TGCTs from TNGCTs and SGCTs from NSGCTs, proving that T2-weighted based radiomics is a promising tool for the diagnostic workup of testicular tumors. The publication of this dataset could provide the means for an independent validation of an AI-based model developed by other research center^12^.

## Methods

### Study population and eligibility criteria

A dataset of MR images from 42 patients with testicular cancer was analyzed. In compliance with current legislation, the research was carried out following approval of our institute’s Internal Review Board.

**Fig. 1:**
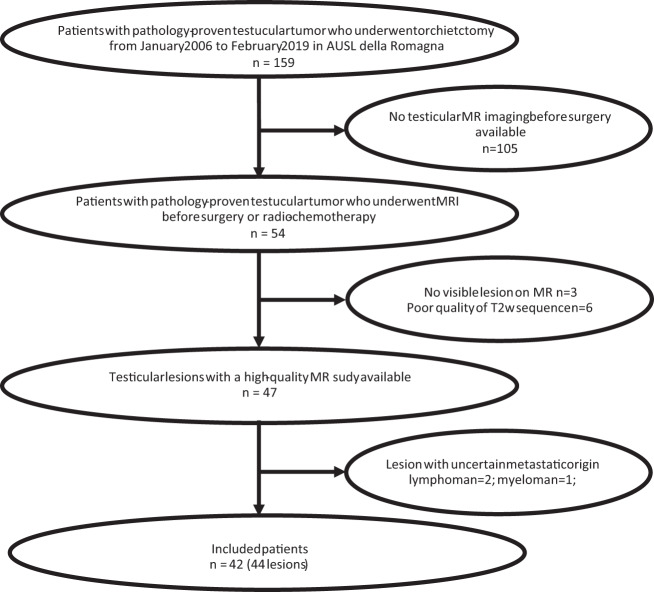
Flowchart of the patient recruiting process.

We identified patients submitted to biopsy/orchiectomy from January 2006 to February 2019 and for whom a histological diagnosis of testicular cancer was subsequently confirmed by the Pathology Unit of Morgagni-Pierantoni Hospital, Forlì (Forlì Local Health Authority - *Azienda USL della Romagna*, Forlì), Italy. Patients for whom T2- and T1-weighted imaging was available in our imaging archive system (*i.e*. Carestream VuePACS, Carestream Health, Rochester, NY, USA) were selected. Exclusion criteria were as follows: (*a*) patients who underwent MRI after surgery or radiotherapy and/or chemotherapy; (*b*) poor quality of MR images due to movement artefacts; (*c*) lesion not visible on MRI; (*d*) metastatic tumor (Fig. 1). In clinical practice, MRI was performed as a second-level problem-solving technique when US results were equivocal/inconclusive, or to obtain detailed local staging of a testicular lesion previously identified by US.

Patient age ranged from 7 to 79 years (mean 39.3 ± 14.3 yrs). One patient had a bilateral classic seminoma and one had 2 different neoplasms, diagnosed years apart. We excluded 2 patients with testicular lymphoma and one with testicular localization of myeloma because of the uncertain origin of the primary tumor (potential metastatic origin of the tumor); another patient with classic seminoma was excluded due to poor image quality. Thus, the final dataset consisted of 42 patients.

### Patient demographics

We analyzed MR studies of 44 testicular lesions (patient and lesion characteristics are summarized in Table 1). The time interval between MRI and final histologic diagnosis was 25 ± 15 days. Thirty-two of the 44 lesions were histologically classified as TGCTs, specifically 23 classic seminomas and 9 NSGCTs (7 mixed germ cell tumors and 2 embryonal cell carcinomas). Twelve of the 44 lesions were TNGCTs or other histological types, of which 7 Leydig cell tumors, 2 Sertoli cell tumors, 2 adenomatoid tumors and one epidermoid tumor. Laterality (left/right) and size were taken into consideration for each lesion. TGCTs were staged according to the 8^*th*^ *Edition of the American Joint Committee on Cancer (AJCC) Staging Manual*^13^.

**Table 1:** Patient demographics and lesion features.

**Germ cell tumors**		
AGE (years)	Average ± standard deviation	36.8 ± 9
LATERALITY	Right/Left	19/13
SIZE (maximum diameter -cm)	Average ± standard deviation	3.2 ± 2.4
STAGING (T)	pT1/pT2/pT3/pT4	17/13/2/0
**Non germ cell tumors**		
AGE (years)	Average ± standard deviation	391 ± 18.6
LATERALITY	Right/Left	4/8
SIZE (maximum diameter - cm)	Average ± standard deviation	0.94 ± 0.46

### MRI T1- and T2-weighted sequence data

All MR T2- and T1-weighted sequences were acquired with the same 1.5 T Scanner (Achieva Philips, Philips Healthcare, Best, Netherlands) using a surface coil (Philips Sense Flex Medium coil). Sequences were acquired with the patients in a feet-first supine position. The surface coil was positioned over a towel covering the scrotum and the penis was dorsiflexed against the lower abdominal wall and taped in place to prevent motion. Peripheral venous access (19-gauge) was obtained in an antecubital fossa vein. The MRI imaging protocol included T1-weighted (T1w), T2-weighted (T2w) and, in some cases, also DWI sequences. Table 2 summarizes the acquisition settings of the scanner including pixel spacing, slice thickness, average echo time (TE), pulse repetition time (TR), and flip angle. Image stored pixel values (SV) are unsigned integers and can be converted through a DICOM reader into real world values (RV) through the Look-up Table Eq. (1):

1 $${\rm{RV}}=({\rm{Real}}\,{\rm{World}}\,{\rm{Value}}\,{\rm{Slope}})\ast {\rm{SV}}+{\rm{Real}}\,{\rm{World}}\,{\rm{Value}}\,{\rm{Intercept}}$$

where Real World Value Slope corresponds to the DICOM tag (0040,9225) or (0028,1052) and Real World Value Intercept to (0040,9224) or (0028,1053), except for the datasets with ID 012, 016, 020, 024, 045.

**Table 2:** Scanner acquisition parameters.

Acquisition Parameter	T1- weighted (mean ± SD)	T2- weighted (mean ± SD)
Slice thickness	3.2 ± 0.3 mm	3.2 ± 0.4 mm
Repetition time	5083 ± 48 ms	4300 ± 1130 ms
Echo time	123 ± 2 ms	105 ± 19 ms
Flip angle	90°/120°	90°/120°
Resolution	0.6 ± 0.1 mm	0.5 ± 0.2 mm

### Manual segmentation of regions of interest

**Fig. 2:**
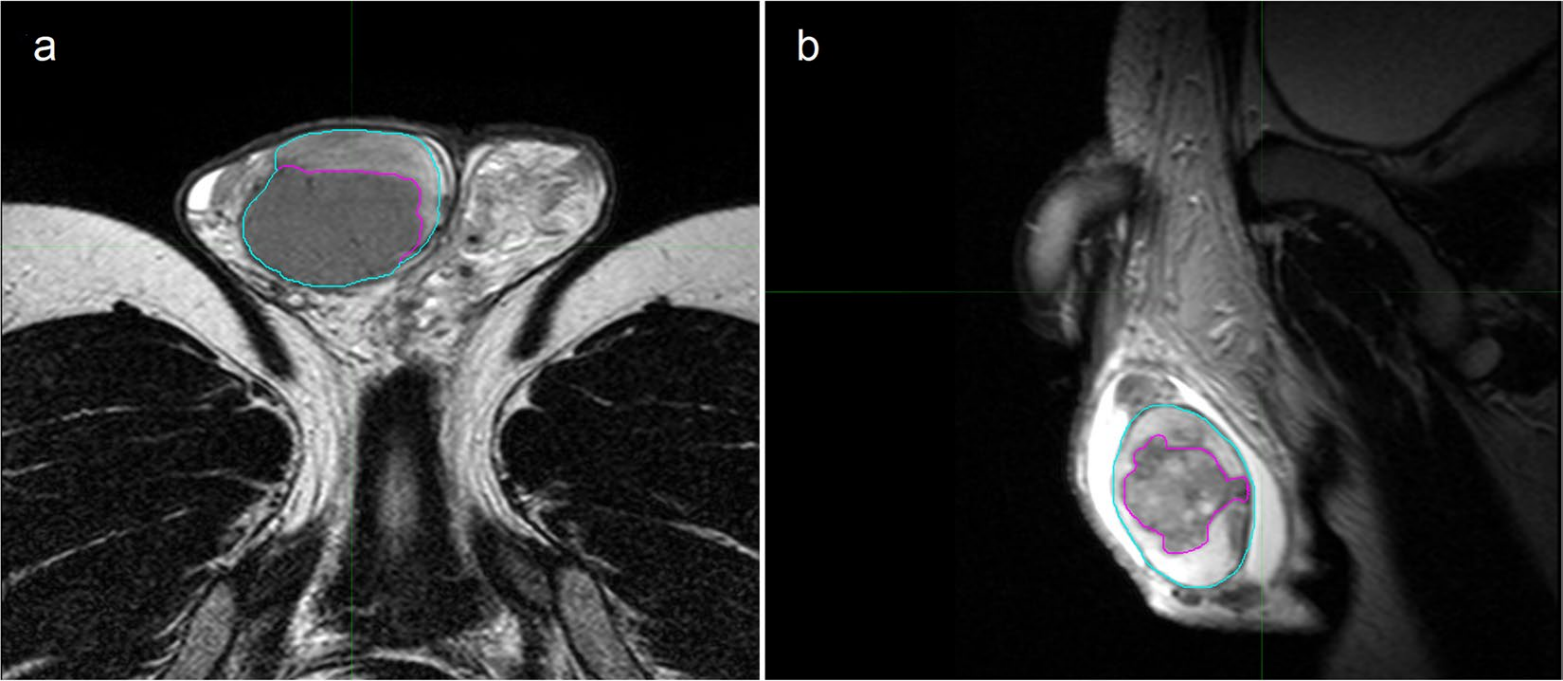
Examples of testicular lesions. (**a**) Testicular seminoma and (**b**) Germinal tumor. Axial and sagittal T2-weighted images, respectively. Testicles are contoured in cyan, whereas neoplasms are contoured in violet.

The patient’s testes and relative lesion were contoured on the T2w sequences (Fig. 2a,b) after joint consensus was reached by two expert radiologists. *MIM Maestro* (MIM Software Inc., Cleveland, OH, USA) was used for contouring.

### Assessment of visual features

During the contouring session, several visual features were also analyzed and are reported in Table 3. The visual features were selected following the indications reported in the work by Tsili *et al*.^7^. The 6 visual properties are as follows: (1) HOMO: this refers to signal homogeneity; (2) LOW SI: relative intensity of the lesion compared to normal testicular parenchyma on T2w sequences; 3) NECRO/HEMO: presence of necrotic or hemorrhagic areas; (4) CAPSULE: refers to the presence of capsule; (5) SEPTA and (6) CE: both refer to the presence and contrast uptake of bandwise regions in T2w sequences.

**Table 3:** Patient clinical data and visual features of the lesions.

ID	AGE	STAGE	HOMO	LOW SI	NECRO/HEMO	CAPSULE	SEPTA	CE
**NON GERMINOMAS (TNGCTs)**								
T010	51	nd	0	0	0	0	0	0
T016	33	nd	0	0	0	0	0	0
T018	22	nd	0	1	1	1	0	0
T019	30	nd	0	1	0	1	0	0
T020	40	nd	1	0	0	1	0	0
T022	63	nd	0	1	0	1	0	0
T023	67	nd	1	1	0	0	0	0
T027	35	nd	0	1	0	1	0	0
T030	46	nd	1	1	0	0	0	0
T039	7	nd	1	1	0	0	0	0
T041	58	nd	0	1	0	1	0	0
T044	12	nd	0	0	0	1	0	0
**Ratio 1** ***vs*** **total (%)**			33	67	8	58	0	0
**GERMINOMAS - NON SEMINOMAS (NSGCTs)**								
T017	37	pT1a	0	1	0	0	1	1
T024	26	pT2	0	0	1	0	0	0
T025	24	pT2	0	1	1	0	0	0
T028	41	pT3	0	1	1	0	0	0
T031	32	pT1	0	1	1	1	0	0
T036	26	pT1	0	0	1	0	0	0
T038	26	pT2	0	0	1	0	0	0
T040	32	pT2	0	1	1	1	0	0
T048	46	pT2	0	0	1	0	0	0
**Ratio 1** ***vs*** **total (%)**			0	55	89	22	11	11
**SEMINOMAS (SGCTs)**								
T001	37	pT2	0	1	1	0	1	1
T002	43	pT1b	0	1	1	0	1	1
T004	54	pT2	0	1	1	0	1	1
T005x	38	pT1b	0	1	0	0	1	1
T005y	38	pT1a	1	1	1	1	0	0
T006	31	pT1a	0	1	0	0	0	0
T007	36	pT1a	1	1	1	0	1	1
T008	37	pT2	1	1	0	0	0	0
T009	39	pT1b	1	1	0	0	1	1
T011	60	pT3	0	1	0	0	1	1
T012	26	pT1a	0	1	0	0	1	1
T013	36	pT1a	1	1	0	1	0	0
T015	50	pT2	0	1	1	0	1	1
T021	44	pT2	0	0	1	0	1	1
T029	31	pT1a	1	1	0	0	1	1
T032	30	pT1a	0	1	0	0	1	1
T033	35	pT1a	0	1	0	0	0	0
T034	44	pT2	0	1	1	0	1	1
T035	23	pT1b	1	1	0	0	1	1
T037	40	pT2	0	1	0	0	0	0
T042	35	pT1b	0	1	0	0	1	1
T045	49	pT1a	0	1	1	1	1	0
T047	43	pT2	0	1	0	0	0	0
**Ratio 1** ***vs*** **total (%)**			30	95	39	13	70	65

TNGCTs stage is marked as not defined (nd). The presence of a certain characteristic in the lesions is labelled by (1) HOMO: refers to signal homogeneity; (2) LOW SI: relative intensity of the lesion compared to normal testicular parenchyma on T2w sequences; (3) NECRO/HEMO: presence of necrotic or hemorrhagic areas; (4) CAPSULE: refers to the presence of capsule; (5) SEPTA and (6) CE: both refer to the presence and contrast uptake of bandwise regions in T2w sequences.

### De-identification of imaging data

All of the images in the dataset were cropped to preserve only the patient’s anatomical details related to the region of interest. Furthermore, in compliance with the current legislation on privacy, we de-identified and coded each patient with an unique ID.

### Tumor surgery and clinical endpoint evaluation

Radical inguinal orchiectomy with removal of the tumor-bearing testis and the spermatic cord up to the inner inguinal ring is the gold standard for diagnosis and local treatment of patients with testicular malignancies. Furthermore, a biopsy is performed on a frozen section of the histological material if the diagnosis of testicular cancer is still not certain. These procedures determine the definitive histopathological classifications reported in our dataset. Further details on orchiectomy can be found in the work by Ghoreifi *et al*.^14^.

## Data Records

We created a public *figshare* collection called “2021_FelicianiGiacomo_Collection1”^15^ containing:

- Original MRI images: T1-weighted (T1w) and T2-weighted (T2w) MRI images in DICOM format referring to the scrotal region of patients with testicular tumors.
- Contouring RadioTherapy Structure Set (RTSS): Manual contouring in DICOM format, performed by the expert radiologists who analyzed the testis and associated lesion.
- Summary of patient characteristics: Excel Table reporting the demographic, clinical and visual characteristics of patients.

The collection is publicly available at: 10.6084/m9.figshare.c.5277818.v1.

## Technical Validation

All MRI data were collected as part of routine patient healthcare and thus quality assurance was performed by *Azienda USL della Romagna*, where the data were collected.
